# A Combined In Vitro Imaging and Multi-Scale Modeling System for Studying the Role of Cell Matrix Interactions in Cutaneous Wound Healing

**DOI:** 10.1371/journal.pone.0148254

**Published:** 2016-02-03

**Authors:** Aribet M. De Jesus, Maziar Aghvami, Edward A. Sander

**Affiliations:** Department of Biomedical Engineering, University of Iowa, Iowa City, IA, United States of America; Université de Technologie de Compiègne, FRANCE

## Abstract

Many cell types remodel the extracellular matrix of the tissues they inhabit in response to a wide range of environmental stimuli, including mechanical cues. Such is the case in dermal wound healing, where fibroblast migrate into and remodel the provisional fibrin matrix in a complex manner that depends in part on the local mechanical environment and the evolving multi-scale mechanical interactions of the system. In this study, we report on the development of an image-based multi-scale mechanical model that predicts the short-term (24 hours), structural reorganization of a fibrin gel by fibroblasts. These predictive models are based on an *in vitro* experimental system where clusters of fibroblasts (i.e., explants) were spatially arranged into a triangular geometry onto the surface of fibrin gels that were subjected to either *Fixed* or *Free* in-plane mechanical constraints. Experimentally, regional differences in short-term structural remodeling and cell migration were observed for the two gel boundary conditions. A pilot experiment indicated that these small differences in the short-term remodeling of the fibrin gel translate into substantial differences in long-term (4 weeks) remodeling, particularly in terms of collagen production. The multi-scale models were able to predict some regional differences in remodeling and qualitatively similar reorganization patterns for the two boundary conditions. However, other aspects of the model, such as the magnitudes and rates of deformation of gel, did not match the experiments. These discrepancies between model and experiment provide fertile ground for challenging model assumptions and devising new experiments to enhance our understanding of how this multi-scale system functions. These efforts will ultimately improve the predictions of the remodeling process, particularly as it relates to dermal wound healing and the reduction of patient scarring. Such models could be used to recommend patient-specific mechanical-based treatment dependent on parameters such as wound geometry, location, age, and health.

## Introduction

Cutaneous wound healing involves the coordination of platelet degranulation, provisional fibrin matrix formation, cellular infiltration, and extracellular matrix (ECM) remodeling, and continues for a period after skin integrity and homeostasis is restored [1]. The repaired tissue formed is fibrotic (i.e., a scar) and lacks the organization and full functionality of normal skin. Additionally, if imbalances between ECM synthesis and degradation arise during the remodeling process, it can lead to abnormal scars, such as hypertrophic scars, that are characterized by excessive fibrosis. These scars can result in disfigurement, distress, discomfort/pain, and permanent loss of function from contracture [2–4]. Abnormal scarring is a major clinical problem, with estimated U.S. annual treatment costs in the billions of dollars [5,6].

A number of clinical treatments have been explored to manage these scars, including surgical excision, corticosteroid injection, silicone gel sheeting, pressure therapy, and laser therapy [3]. For many of these treatments, the mechanisms underlying a reduction in fibrosis (as well as the range in patient healing response) are not clear. There is, however, increasing evidence supporting the notion that the improvement in scar formation observed with these treatments has a mechanical basis, particularly as fibrosis is believed to be a response to tension [6–8], and these treatments may all act to reduce tension during healing [9–11]. The idea that mechanical environment plays an important role in scar management is also consistent with the surgical practice of making incisions in the skin along Langer’s lines to reduce tension and scarring at the wound site. Furthermore, wounds formed in high-tension areas, such as the shoulder and scapula, are more prone to exuberant fibrosis than low-tension areas, such as the scalp and lower legs [7].

*In vitro* experiments have also demonstrated that mechanical forces modulate the activity of skin-derived cells [12–15], including fibroblast gene expression for inflammatory mediators, growth factors, and ECM proteins [16–19]. Additionally, *in vivo* studies on mice, pigs, and humans have shown that altering skin stress dramatically regulates the extent of scarring [20–22]. Recently, Gurtner et al. found that stress shielding abdominal incisions produced significant improvements in scarring compared to within-patient controls [21,23]. Other important mechanisms are also involved (e.g. oxygen tension, inflammatory agents, biochemical and genetic factors) but clearly there are substantial data supporting a central role for mechanical environment.

Multi-scale mechanical interactions are an important component of this mechanical environment. They are dynamic and reciprocal scale-spanning physical interactions between tissues, cells, and ECM. In the wound site these interactions initially involve fibrin and the structure of the clot, and then gradually change as fibrin is replaced with new ECM, like load-bearing collagen. When loads and constraints are applied at the tissue-level of the skin, they are transmitted down into the provisional matrix and ECM, where they can alter the local environment and affect cell activity. In addition, the constituent fibroblasts themselves also exert traction forces on the ECM in an attempt to close the wound margins and to reach a homeostatic level of ECM tension. Combined, these interactions contribute in an integrated and cooperative manner to produce a global pattern of ECM remodeling [20,24–26], including during wound healing and scar formation [20,27].

It is, however, difficult to evaluate what distinguishing parameters from the complex milieu of components involved result in fibrosis, or what measures should be taken to minimize scar formation. Although it is clear that reducing tension at the wound site can reduce scar formation, it is not clear what mechanobiological mechanisms mediate this process or how it can be optimized. With many complicated phenomena operating at multiple scales and governed to varying degrees by the properties of the ECM, mechanical models become a necessary tool for unraveling the relationships between individual ECM and cellular components and the aggregate remodeling response and properties of the healing tissue. As such, these models will be important for evaluating parameters unique to a patient and identifying an optimized strategy for reducing scar.

In addition, while much of the focus on mechanical environment has rightly been placed on the levels of tension at the wound site, a second component involved in multi-scale mechanical interactions that is potentially important is the initial configuration of the clot. This initial structure may control both how macroscopic forces are distributed through the microstructure (and thus the levels of force an individual cell will experience) and how replacement ECM (e.g. collagen, elastin) will be organized; organization will be controlled partly through cell contact guidance with the fibrin matrix and steric constraints on how ECM can be laid down and assembled [28–30]. Recent work suggests that the remodeling response in an *in vitro* setting is strongly influenced by multi-scale mechanical interactions that depend in part on the initial fibrin alignment pattern and the distribution of cell-generated internal forces [26]. Should this proposition hold true, knowing how and when one should intervene (e.g. mechanically or chemically) to alter this pattern and control the remodeling process will be important for optimizing treatments to reduce scar. In strategies that involve changing the mechanical environment of the wound site (e.g. stress shielding sheets, shape memory sutures, sutures with elastic gradients, and adhesives), many important variables are not optimally defined. For example, it is not clear if there is an optimal window in time for stress shielding the wound site, how much or what kind of force should be applied, whether the amount of force should change over time, or how these parameters should change with anatomical site, wound size, and shape.

Here, we report on our initial work to develop a system for understanding how multi-scale mechanical interactions regulate wound healing and scar formation so that we can answer these sorts of questions. This system combines an *in vitro* dermal fibroblast explant/fibrin gel culture subjected to time-lapse imaging with a fiber-based multi-scale computation model. Small, but significant, differences in short-term structural remodeling of fibrin gels by the fibroblasts in response to *Fixed* or *Free* gel boundaries were observed that could impact long-term remodeling. In addition, image-based models of the experiments were able to qualitatively predict some features of the remodeling process. This combined system of experiments and image-based models represents a new model system for understanding the mechanobiology of scar formation and for devising and assessing new treatments to manage scar formation.

## Methods

### Fibrin Gel Preparation

Fibrin gels were prepared as described previously [26,31]. Briefly, 0.222 mL of bovine fibrinogen at a concentration of 30.6 mg/mL (Sigma Aldrich, St. Louis, MO) was diluted in 0.444 mL of 20 mM HEPES buffer and combined with 0.167 mL of a solution of bovine thrombin (Sigma Aldrich, St. Louis, MO), and CaCl_2_ to produce 6.8 mg/mL fibrin gels. Microspheres measuring 4 um in diameter (Life Technologies, Grand Island, NY, Catalog No. F8858) were also added at a concentration of 5 million microspheres/mL and homogeneously distributed in the solution before gelation commenced. The solution was then cast into square polydimethylsiloxane (PDMS) (Dow Corning, Midland, MI) molds measuring 8 mm x 8 mm x 1 mm (length, width, depth). The molds were attached with silicone grease (Dow Corning, Midland, MI) to either the No. 0 coverglass of 35 mm glass bottom Petri-dishes (MatTek Corp., Ashland, MA, Catalog No. P35G-0-20-C) or the viewport glass of a microscope-mounted bioreactor (described below). The solution gelled quickly in the mold and remained at room temperature for approximately 30 minutes until the explants were added. This process produced gels with an estimated average thickness of 798 μm ± 139 μm. This measurement was calculated based on multiplying the optical path length (i.e., the difference between the focal plane of the glass surface and focal plane of the gel surface) and the refractive index of the gel, which we took as the refractive index of water, n = 1.3.

### Cell Culture and Explant Preparation

Immortalized TERT-human dermal fibroblasts [32] were cultured in high glucose Dulbecco’s Modified Eagle Medium (DMEM) (Life Technologies, Grand Island, NY) supplemented with 10% fetal bovine serum, 1% penicillin-streptomycin, and 0.1% amphotericin B. Fibroblasts were grown to 80% confluency in T-75 tissue-culture flasks maintained in a 37°C humidified incubator supplied with 5% CO_2_ and 95% air. Explants, each consisting of approximately 6,000 cells, were produced by trypsinizing, pelleting, and resuspending the fibroblasts in a low retention 1.5 mL conical tube at a concentration of 2x10^7^ cells/mL, and pipetting 0.3 μL of cell suspension onto the surface of a fibrin gel. Explants were chosen over homogeneously dispersed single cells because they provide a simplified and convenient system for evaluating cell-driven structural reorganization for simple geometric configurations and boundary conditions that can be easily incorporated into the existent multi-scale model [33]. Three explants were positioned at the vertices of a triangle with each side measuring approximately 2 mm from explant centroid-to-centroid (c.f., [31]). The cells were then allowed to settle and attach to the fibrin gel for approximately 2 hours before fresh medium with 10 μg/mL aprotinin was added in order to limit the potential for serine protease induced fibrin degradation [34].

### Gel Boundary Conditions

**Fig 1:**
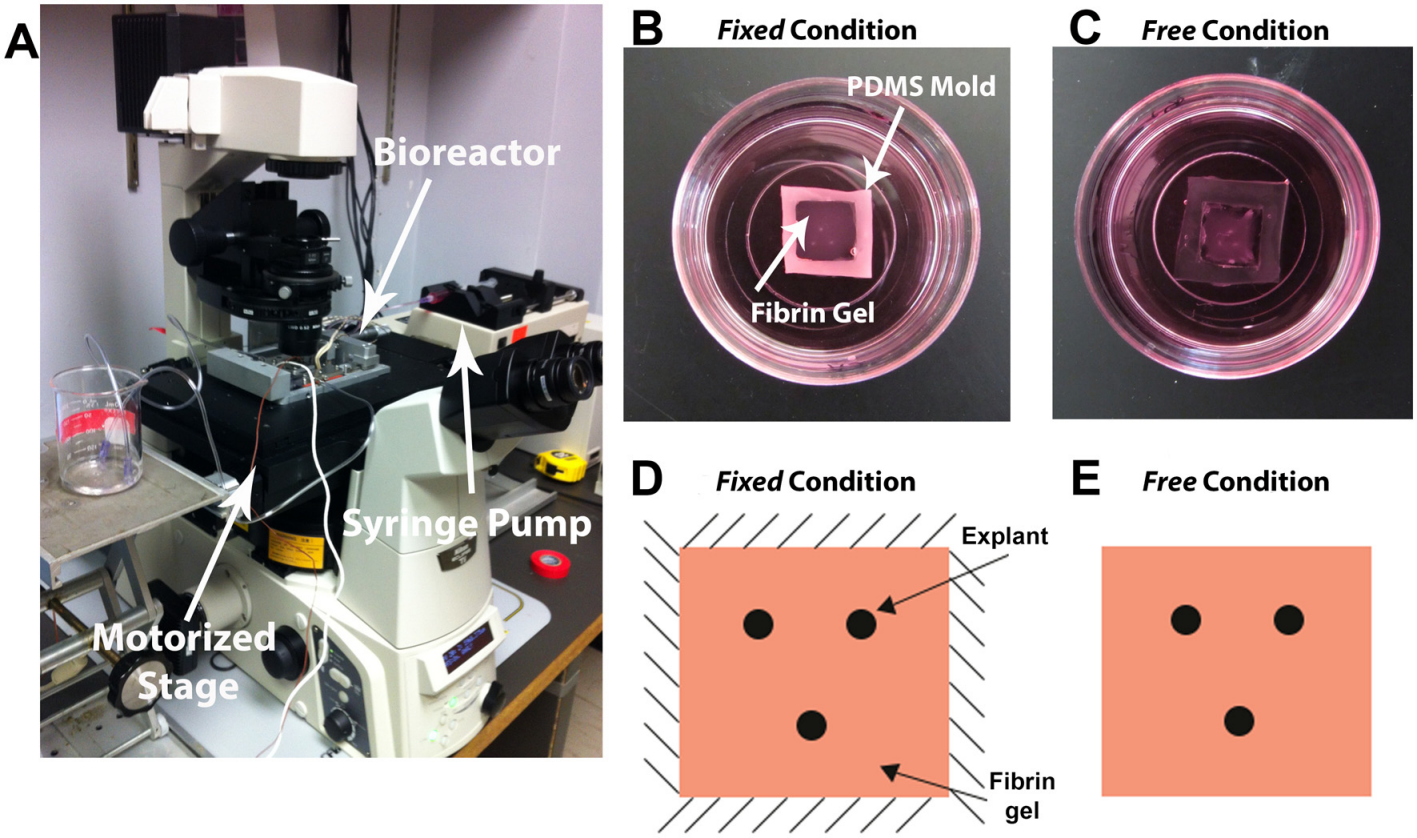
Experimental setup and explant boundary conditions. (A) An inverted Nikon Eclipse Ti microscope operating in DIC mode was used to image the fibrin-explant system. Samples were prepared and maintained in either a microscope-mounted ADMET BioTense Bioreactor (as shown in A) or in glass bottom Petri-dishes (as shown in B and C). (B) Fibrin gels were polymerized in a PDMS mold and explants were placed in a triangular configuration and subjected to either (B, D) *Fixed* or (C, E) *Free* in-plane boundary conditions.

In order to study the effects of mechanical constraints and multi-scale mechanical interactions on explant restructuring of the gel, two in-plane boundary conditions, designated as either *Fixed* or *Free*, were examined (Fig 1). For *Fixed* gels, the sides of the gel remained attached to the PDMS mold. For *Free* gels, the sides of gel were carefully separated from the edges of the mold with a sterile 30G x ½ inch needle (BD Biosciences, San Jose, CA). The gel bottoms remained attached to the glass for both cases.

### Experimental Setup and Imaging

Four samples were prepared for both *Fixed* and *Free* boundary conditions, with each gel cast into its own separate 35 mm glass bottom Petri dish. These samples were transferred from the incubator to the microscope and imaged at t = 0 hours, 12 hours, and 24 hours in order to provide an indication of experiment repeatability, which was assessed by monitoring gross structural changes in the gel, such as the changes in explant area and explant centroid-to-centroid distance, and to confirm that suitable environmental conditions were maintained in the bioreactor.

**Fig 2:**
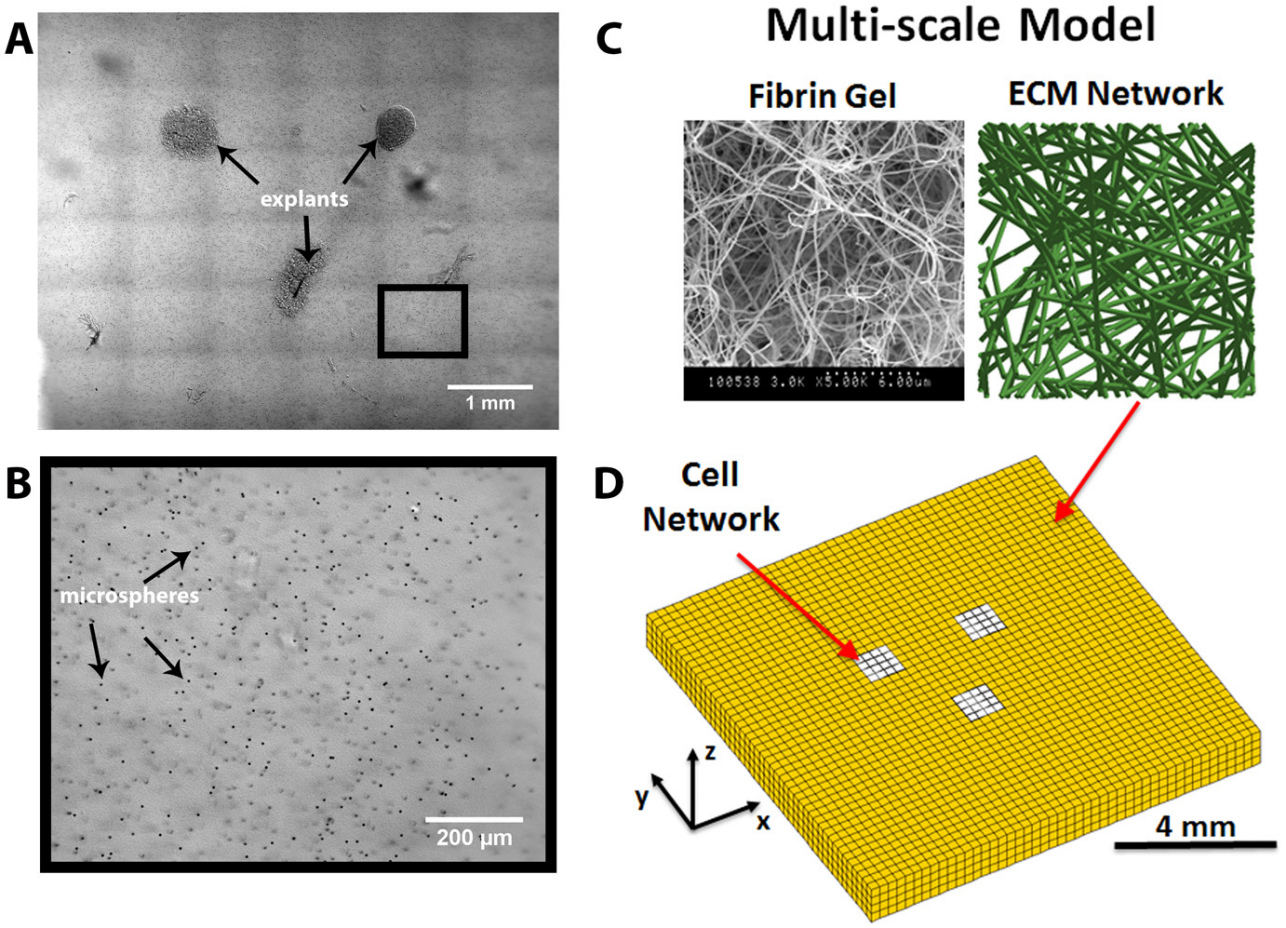
In vitro data collection and image-based computational model. (A) Three explants were placed at a distance of approximately 2 mm from each other to form the vertices of a triangle. A tiled image consisting of 36 individual 10x DIC images was acquired every 15 minutes in order to observe and quantify structural and morphological changes in the fibrin gel. (B) Over the course of the experiment the instantaneous and cumulative displacements of a subset of embedded microspheres was analyzed at each location (the depicted image corresponds to the black box in (A)). (C) The image-based computational model consists of finite elements containing fiber networks that are representative of the fibrin fibers in the gel. (D) The model is partitioned into cellular networks (white elements) and surrounding fibrin networks (yellow elements) that are configured to approximate the geometric configuration of the explants and gel in the experiments.

A more detailed view of structural changes in the gels over 24 hours was also obtained by taking time-lapse images of one *Fixed* and one *Free* gel (each gel was imaged separately) in a temperature-controlled, microscope-mounted BioTense Perfusion Bioreactor (ADMET, Norwood, MA), which was maintained at 37°C (Fig 2A and 2B). Fresh incubator-conditioned medium saturated with 5% CO_2_ was perfused through the bioreactor via a syringe pump at a rate of 0.015 mL/min for the duration of the experiment in order to maintain a pH of 7.4. Outflow medium from the bioreactor was collected and periodically tested with a Combination pH Microelectrode (Cole Parmer, Vernon Hills, IL) to confirm that a neutral pH was maintained. All images were obtained on a Nikon Eclipse TI inverted microscope equipped with a DS-Qi1 Nikon CCD camera and a ProScan II motorized stage. Gels were imaged with a CFI Plan Apo 10x DIC objective. During imaging 36 individual frames were acquired and tiled together. All images were saved as 16-bit jpg2 images and exported as uncompressed 8-bit tiff images for analysis.

Fiber alignment was not clearly visible in these gels with DIC imaging due to light scattering from the microspheres and the thickness of the gel. As a result, fiber alignment was obtained at 24 hours by imaging a set of identical gels (minus the microspheres) fixed with 4% paraformaldehyde. These gels were imaged in reflection mode [35,36] using a Leica TCS SP5 multi-photon confocal microscope with a HeNe 543 nm laser and a 20x objective (Roy J. Carver Center for Imaging, University of Iowa). Cell nuclei were labeled with Hoescht 33342 (Life Technologies, Grand Island, NY). Images were acquired at adjacent locations as a z-stack consisting of 40 images spaced 4 μm apart through the thickness. All images were saved as 8-bit tiff images.

### Image Analysis

#### Changes in Explant Morphological Changes and Cell Migration

Images acquired at t = 0 hours, t = 12 hours, and t = 24 hours were analyzed with ImageJ (NIH, Bethesda, MA) in order to measure morphological changes in explant area and centroid-to-centroid distance. Explants borders were delineated with the free-hand drawing tool in order to calculate the area and centroid of each explant. Cells that migrated past the periphery of each explant were not considered as part of the explant area. The percent change in explant area was calculated by normalizing the explant area at t = 12 hours and t = 24 hours with the area at t = 0 hours. Similarly, the centroid-to-centroid distance between explants was normalized with the corresponding distance between explants at t = 0 hours.

Cell migration distance, *CMD*, out of the explant was also assessed from images acquired at 24 hours. Regional distinctions were made between cells traveling axially between explants versus those moving towards the gel boundaries. This distinction between axial and non-axial regions was made in order to determine if fiber alignment generated between explants resulted in enhanced cell migration, since fiber alignment is known to influence cell migration patterns [28,37,38]. In each region, radial lines were drawn outward from the explant boundary, through the axis of the migrating cell body, and up to the leading edge of the migrating cells. The average line length and standard deviation was then taken as the *CMD* for the region.

#### Microsphere Displacements

For each of the time-lapse images acquired, the position of a subset of microspheres (17 ± 3 microspheres/image) was tracked during the experiment using a custom semi-automated MATLAB algorithm [13,39]. The algorithm, which is based on normalized cross-correlation between successive images, was then applied to each individual image sequence. First, a set of clearly visible microspheres was selected in the initial image of the time-lapse sequence and the instantaneous and cumulative displacements of each microsphere were determined and updated via the template-matching algorithm. Similarly, the instantaneous and period average rates of microsphere displacement were calculated, respectively, by either dividing the difference in microsphere displacement between successive frames by the time between image acquisitions (i.e., 15 minutes) or by dividing the difference in the cumulative displacement from 0 to 6 hours, 6 to 12 hours, 12 to 18 hours, and 18 to 24 hours over the time in minutes (i.e., 360 minutes). Note that for the *Free* gel, microspheres in the images making up the far right column were not tracked because the images were not in focus.

During the experiment cell compaction of the gel produced large deformations in the plane and through the thickness of the gel. To compensate, the focal plane was periodically readjusted over the course of the experiment to keep the microspheres in focus. The accuracy of the calculated displacements was visually confirmed frame-by-frame by plotting the displacement vectors on the tracked microspheres in the image. The microsphere displacements associated with each tiled image were further grouped into one of four spatially distinct regions (S1 Fig) to facilitate identifying regional differences in remodeling. These regions include the non-axial region above the explants (Region 1), the lateral non-axial regions (Region 2), the axial regions and the area in the center of the explant (Region 3), and the non-axial region below the explants (Region 4).

#### Fibrin Fiber Alignment

Fiber alignment at 24 hours was measured in the pair of paraformaldehyde-fixed gels using a custom MATLAB algorithm based on the use of Fast Fourier Transforms [40]. The algorithm returns a fiber orientation distribution that is converted to an orientation tensor with major and minor principal directions of alignment that can also be compared directly with fiber network alignment in the simulations (c.f. Model Formulation and Computational Details). The strength of fiber alignment, α, in the image is then calculated as *α* = 1−*ω*_1_/*ω*_2_, where *ω*_1_ and *ω*_2_ are the eigenvalues of the orientation tensor and *ω*_1_<*ω*_2_. α is bounded by values of 0 and 1, which correspond to isotropic and completely aligned fiber distributions in the image.

### Multiscale Model

Structural reorganization of the fibrin gel by the explants was simulated with a multi-scale modeling technique that has been applied previously to study a range of multi-scale mechanical phenomena, such as collagen gel biomechanics [41–44], enzymatic remodeling [45], tissue failure [46], and cell compaction [33]. In these models, the Galerkin finite element method is used to describe the macroscopic scale of the gel (i.e., geometry and boundary conditions). Representative volume elements (RVE) containing cross-linked fiber networks are used to represent the microscopic scale. Volume averaging theory is then used to couple the scales. As a result, three essential equations are employed in the model: (1) a macroscopic-level stress equilibrium equation; (2) a microscopic-level fiber constitutive equation; (3) and a volume-average stress equation that converts the microscopic forces in the fiber network to a macroscopic stress tensor. A detailed description of these equations and their implementation can be found in previous work [42,44]. Briefly, the expression for the macroscopic stress equilibrium is given as

$$\langle \sigma_{ij,i}\rangle +\sigma_{ij,i}^{nH}=\frac{1}{V}\oint_{\delta V}\left(\sigma_{ij}-\langle \sigma_{ij}\rangle \right)u_{k,i}n_{k}dA$$ (1)

where 〈σ_*ij*_〉 is the macroscopic volume-averaged Cauchy stress of the fiber network, $\sigma_{ij}^{nH}$ is an additive neo-Hookean stress (defined below), σ_*ij*_ is the local microscopic stress, *u* is the displacement of the RVE boundary, and *n* is the unit normal vector to the boundary surface. The components of these stresses are related to the RVE volume, *V*, and the components of the position, *x*_*i*_, and force, *F*_*j*_ of the fiber nodes on the RVE boundary by

$$\langle \sigma_{ij}\rangle =\frac{1}{V}\int_{V}^{}\sigma_{ij}dV=\frac{1}{V}\sum_{boundary\;nodes}x_{i}F_{j}$$ (2)

The force required to deform a fiber is calculated from a phenomenological constitutive equation [47]

$$F=\frac{E_{f}A_{f}}{B}\left[\text{exp}(B\varepsilon_{f})-1\right]$$ (3)

where *E*_*f*_ reduces to Young’s modulus in the limit of small strain, *A*_*f*_ is the cross-sectional area of a fiber, *B* is a fitting parameter that controls the nonlinearity of the force, and $\varepsilon_{f}=0.5\left(\lambda_{f}^{2}-1\right)$ is the Green’s strain defined in terms of the fiber stretch ratio, *λ*_*f*_. In order to limit the compressibility and distortion of the fibrin fiber networks, a continuum-level compressible neo-Hookean stress was added to the volume-averaged stress (c.f. [48]). This stress is given as

$$\sigma_{ij}^{nH}=\frac{G}{J}\left(B_{ij}-\delta_{ij}\right)+\frac{2G\nu }{J\left(1-2\nu \right)}\left(\text{ln}\left(J\right)\right)\delta_{ij}$$ (4)

where *G* is the shear modulus, *v* is Poisson’s ratio, *J* is the determinant of the deformation tensor, and *B* is the left Cauchy-Green deformation tensor [49].

### Model Formulation and Computational Details

Previously, we setup a multi-scale model of explant compaction of a collagen gel in which the FE domain was partitioned into cellular and extracellular matrix (ECM) domains [33]. The effect of cell traction forces on the structural reorganization of the surrounding matrix was assessed by incrementally shortening the reference length of all the fibers in the cellular networks. The procedure generated tensile force in the cellular domain that induced substantial reorganization in the ECM networks in a manner dependent on the geometry of the problem. Here, image-based models were produced for direct comparison with the experiment as follows. First, a square FE mesh was created that matched the fibrin gel’s dimensions of 8 mm x 8 mm x 0.8 mm (length, width, depth) and approximated the average geometric spacing and size of the triangular explants (Fig 2C and 2D). The mesh, which contained 46 elements along the length, 46 elements along the width, and four elements through the depth, consisted of 8,464 tri-linear hexahedral elements and 11,045 nodes (Fig 2). It was partitioned into a cellular domain consisting of three triangularly arranged squares, each consisting of 16 adjacent surface elements (i.e., one element through the depth), and a surrounding ECM domain. The model consisted of a collection of nearly isotropic 3D fiber networks that contained on average 337 ± 30 cross-linked fibers. Since experimental data about fiber realignment through the thickness was not available, network orientation and strength of alignment was quantified in a manner consistent with previous work using the 2D projections of the 3D microstructure as

$$\Omega =\frac{\sum_{i=1}^{NF}l_{i}\left[\begin{array}{cc} {\text{cos}}^{2}\;\theta_{i} & \text{cos}\;\theta_{i}\text{sin}\;\theta_{i}\\ \text{cos}\;\theta_{i}\text{sin}\;\theta_{i} & {\text{sin}}^{2}\;\theta_{i} \end{array}\right]}{\sum_{i=1}^{NF}l_{i}}$$ (5)

where Ω is the length-weighted 2D orientation tensor, *θ*_*i*_ is the angle that fiber *i* makes with the horizontal axis, *l*_*i*_ is the length of fiber *i*, and *NF* is the number of fibers in the network. The eigenvectors and eigenvalues of this tensor represent the principal directions and magnitudes of fiber orientation in the network, respectively, and the strength of alignment, α, is defined the same as in Fibrin Fiber Alignment.

Image-based models of the experiment were constructed by using the average explant area and the centroid-to-centroid distances (*Free* and *Fixed* gels combined) at the start of the experiment as target values for the model. These values were not matched exactly due to the discrete nature of the elements and computational constraints on increasing the number of elements. As a result, the simulated explant area was given a value of 0.484 mm^2^ compared to an average initial explant area in the experiments of 0.463 ± 0.089 mm^2^ (average of both *Free* and *Fixed* cases). Similarly, the horizontal centroid-to-centroid distance, which was larger between the top two explants, was set at 2.09 mm compared to 2.2 ± 0.15 mm in the experiment. The centroid-to-centroid spacing of the other two axes were set to 2.03 mm in the model compared to a spacing of 2.03 ± 0.12 mm and 1.93 ± 0.21 mm in the experiment.

To simulate the *Fixed* experiment, all FE mesh surfaces except the top surface, which remained free, were fixed. To simulate the *Free* experiment, the only constraint on the FE mesh was on the bottom surface, where the nodes contained in an area at the center measuring 1.74 mm x 1.74 mm were fixed. The surrounding elements were allowed to translate inward in a manner consistent with the small reduction (< 2%) in gel area observed experimentally, where the gel became partially detached from the substrate around the periphery. Structural remodeling due to explant traction forces was simulated by incrementally reducing the reference lengths of all of the fiber in the cellular networks uniformly by 24%. This value was selected so that the simulated final average area of an explant matched the average measured area of the explants for the *Free* boundary condition after 24 hours in the experiments. The *Free* condition was selected because the decrease in explant area was slightly greater in the *Free* boundary case than in the *Fixed* boundary case. Both cell and gel networks were assigned values of *E*_*f*_*A*_*f*_ = 3*x*10^-10^*N* and *B* = 4, and *G* = 1 Pa and *v* = 0.3 for the neo-Hookean component. These values were obtained by fitting a model to the stress-strain curve generated from a uniaxial mechanical test of a rectangular 6.8 mg/ml fibrin gel.

The computational costs associated with the simulations were very large. There were roughly 1,000 degrees of freedom per fiber network and 67,712 networks in each mesh. As a result, simulations were run using a custom parallelized C code with message passing interface (MPI) on high performance computing resources (ITS Helium Cluster, University of Iowa). Simulations were executed on a single 12-core node (Intel Xenon processor with 144 GB of memory and Infiniband connectivity). The fixed boundary condition simulation required a CPU time and wall time of 282 hours and 58 hours, respectively. The CPU time and wall time for the free boundary condition simulation were 348 hours and 74 hours, respectively.

### Statistical Analysis

All data are presented as mean values ± standard deviation except where noted. Statistical significance (p < 0.05) was determined by performing a two-way analysis of variance (ANOVA) with boundary condition type and time or region as cofactors. Bonferroni post hoc tests were used whenever the outcome of an ANOVA showed statistical significance. All statistical analysis was conducted in MATLAB (Mathworks, Natick, MA).

## Results

### Experiments: Explant Morphological Changes

**Fig 3:**
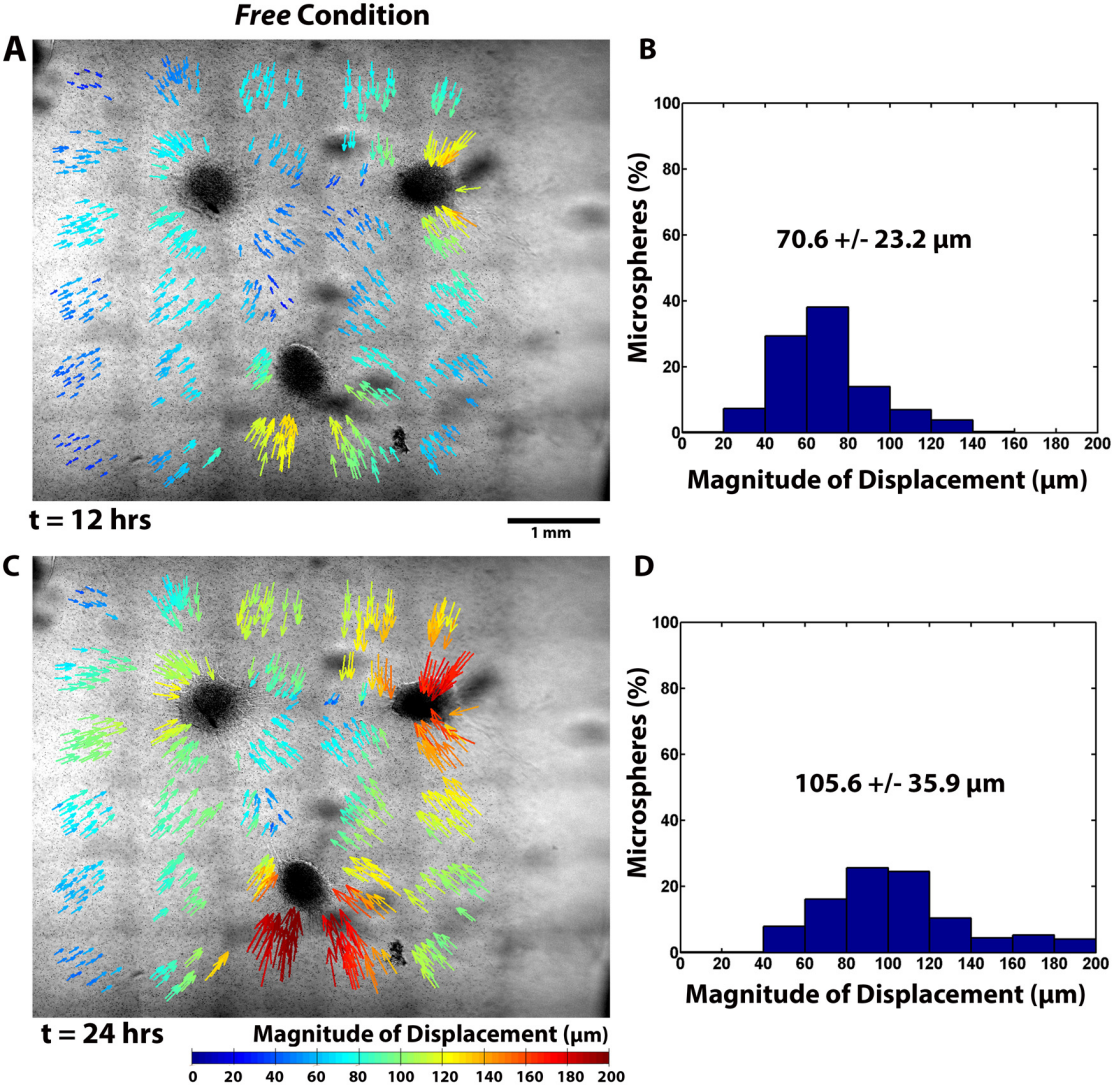
Microsphere displacements in a *Free* gel. Cumulative microsphere displacements at (A,B) t = 12 hours and (C,D) t = 24 are depicted spatially with color coded arrows that indicate the direction and magnitudes. Also shown are the corresponding histograms of microsphere displacement.

**Fig 4:**
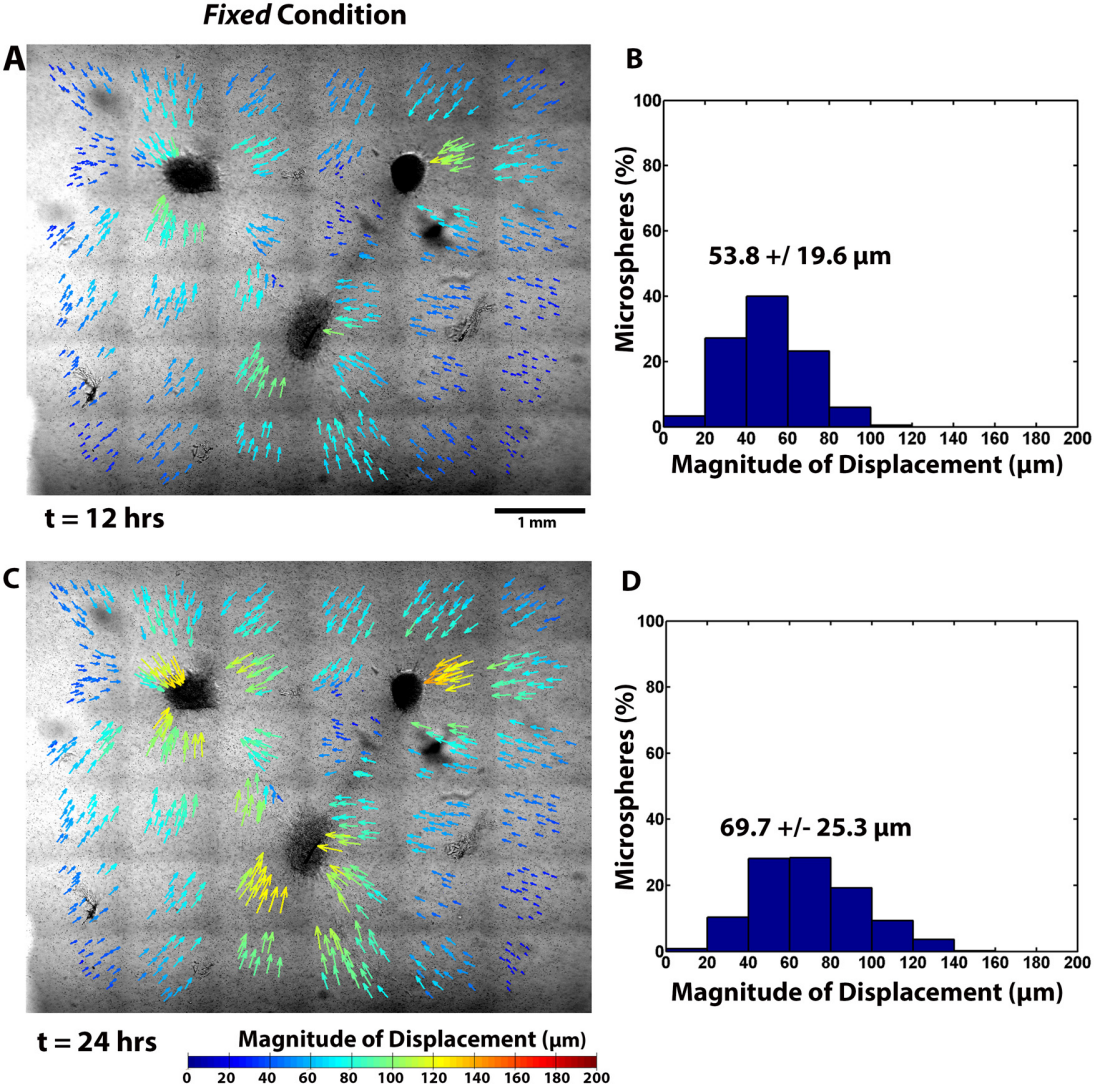
Microsphere displacements in a *Fixed* gel. Cumulative microsphere displacements at (A,B) t = 12 hours and (C,D) t = 24 are depicted spatially with color coded arrows that indicate the direction and magnitudes. Also shown are the corresponding histograms of microsphere displacement.

In all experiments, initially rounded fibroblasts in the explants rapidly reorganized the shape of the explants during the first 6 hours. Over the next 18 hours, the dense mass of cells in the explants continued to shrink in size while individual fibroblasts on the explant periphery began to assume a spindle-shaped morphology and migrate outward from the explant boundary into the fibrin matrix. Small differences in explant morphology were evident between *Free* and *Fixed* gels (Figs 3 and 4, S1 and S2 Movies). Average initial explant area decreased from 0.47 mm^2^ ± 0.01 mm^2^ at *t* = 0 hours to 0.31 mm^2^ ± 0.07 mm^2^ at *t* = 24 hours in *Free* gels, and from 0.46 mm^2^ ± 0.08 mm^2^ at *t* = 0 hours to 0.33 mm^2^ ± 0.12 mm^2^ at *t* = 24 hours in *Fixed* gels. These values correspond to explant areas that were 66.6% ± 17.9% and 70.9% ± 19.9% of the initial explant areas, respectively. Although explant area decreased more in *Free* gels compared to *Fixed* gels, the difference was not statistically significant.

The explants also moved closer to each other in a manner dependent on the boundary conditions. The average normalized centroid-to-centroid distance between explants after 24 hours was slightly lower (but significantly so, p < 0.05) in *Free* gels (0.95 ± 0.02) compared to *Fixed* gels (0.98 ± 0.02). On average, the explants on *Free* gels moved approximately 100 μm closer together, with the average centroid-to-centroid distance decreasing from 2.16 mm ± 0.20 mm at *t* = 0 hours to 2.03 mm ± 0.20 mm at *t* = 24 hours. In contrast, explants on *Fixed* gels remained separated by essentially the same distance (1.96 mm ± 0.24 mm at *t* = 0 hours and 1.95 ± 0.18 mm at *t* = 24 hours).

Small regional differences in fibroblast migration out of the explants were also observed. At *t* = 24 hours, *CMD* values were significantly higher (p < 0.05) for cells migrating in axial regions between explants than for cells migrating in non-axial regions in both *Fixed* (291 μm ± 95 μm and 252 μm ± 79 μm) and *Free* gels (268 ± 94 μm and 206 ± 61 μm), respectively. Significant differences (p < 0.05) in *CMD* between *Fixed* and *Free* gels were only found for fibroblasts located in the non-axial regions.

### Experiments: Fibrin Structural Reorganization

In addition to changes in explant morphology, substantial structural reorganization in the gels was observed and quantified over the duration of the experiment via the displacement of embedded 4 μm diameter microspheres. Long-range communication of cell traction forces between explants, the ECM, and the gel boundaries produced significant spatial and temporal differences in microsphere displacements between *Free* and *Fixed* gels (Figs 3 and 4). Overall in-plane average microsphere displacements (Table 1) were significantly affected by the boundary conditions (p < 0.001), with substantially larger average cumulative displacements occurring in the *Free* gel.

**Table 1:** Overall Average Magnitude and Rate of Microsphere Displacement.

Time	Magnitude (μm)		Time	Rate of Displacement (μm/min)	
	*Fixed*	*Free*		*Fixed*	*Free*
**6 hours**	36.6 ± 12.7	43.6 ± 14.9	**0–6 hours**	0.10 ± 0.04	0.12 ± 0.04
**12 hours**	53.8 ± 19.6	70.6 ± 23.2	**6–12 hours**	0.05 ± 0.02	0.07 ± 0.03
**18 hours**	63.7 ± 23.5	92.2 ± 30.9	**12–18 hours**	0.03 ± 0.02	0.06 ± 0.02
**24 hours**	69.7 ± 25.3	105.6 ± 35.9	**18–24 hours**	0.02 ± 0.01	0.04 ± 0.02

**Fig 5:**
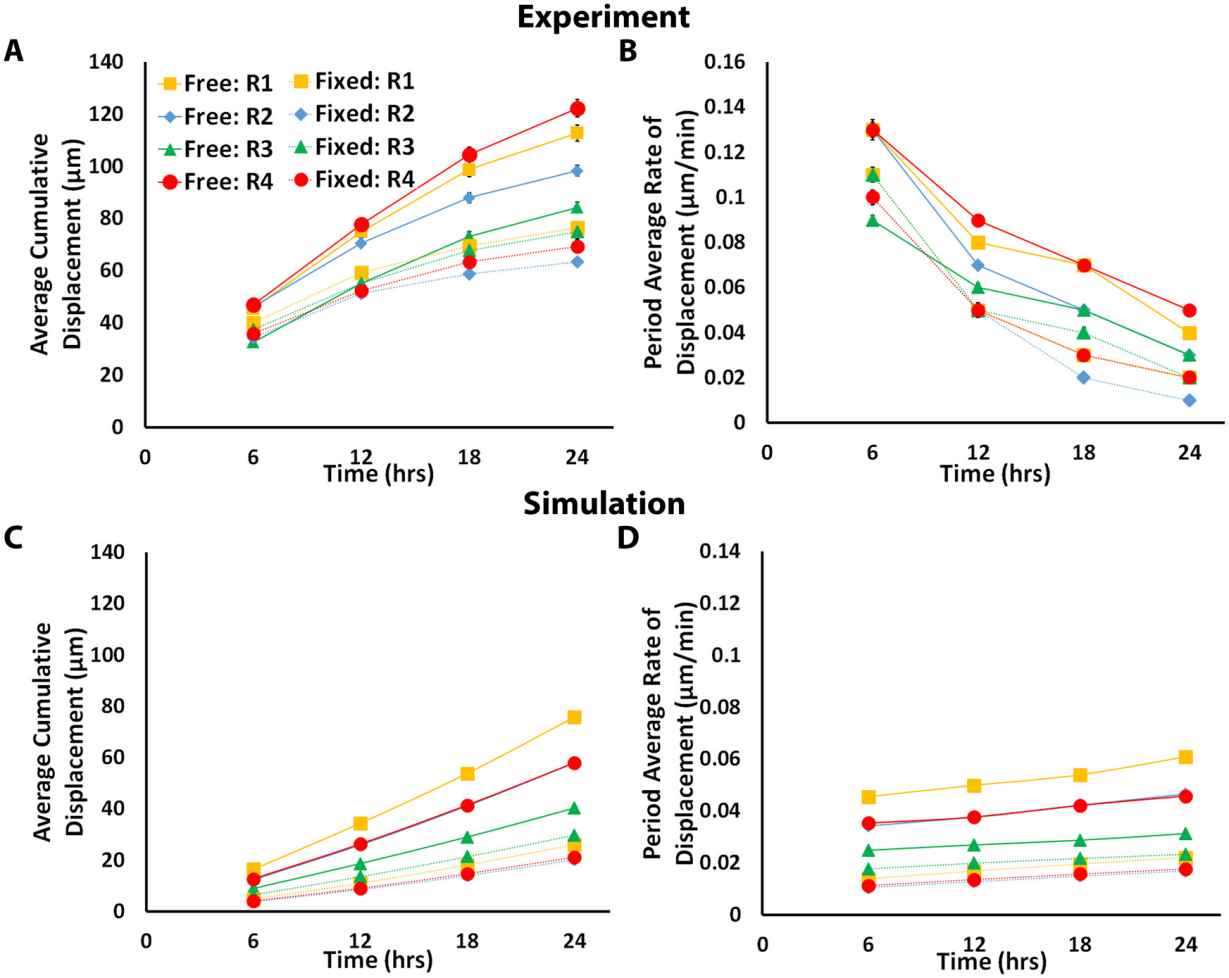
Comparison of regional average magnitude and period average rate of microsphere displacement for each boundary condition and between the experiment and model. (A) Average cumulative microsphere displacement and (B) period average rate of microsphere displacement in the experiment for all four regions and for each boundary condition plotted every six hours. Error bars represent the standard error of the mean. Equivalent plots for the model show the (C) average cumulative FE nodal displacement, and (D) average rates of nodal displacement for all four regions in the model for each boundary condition. The model predicts larger displacements and rates of displacement in the *Free* gel compared to the *Fixed* gel, as was observed experimentally. However, instead of the experimentally observed decrease in the displacement rates with time, the model predicts a gradually increasing rate of displacement with time.

**Fig 6:**
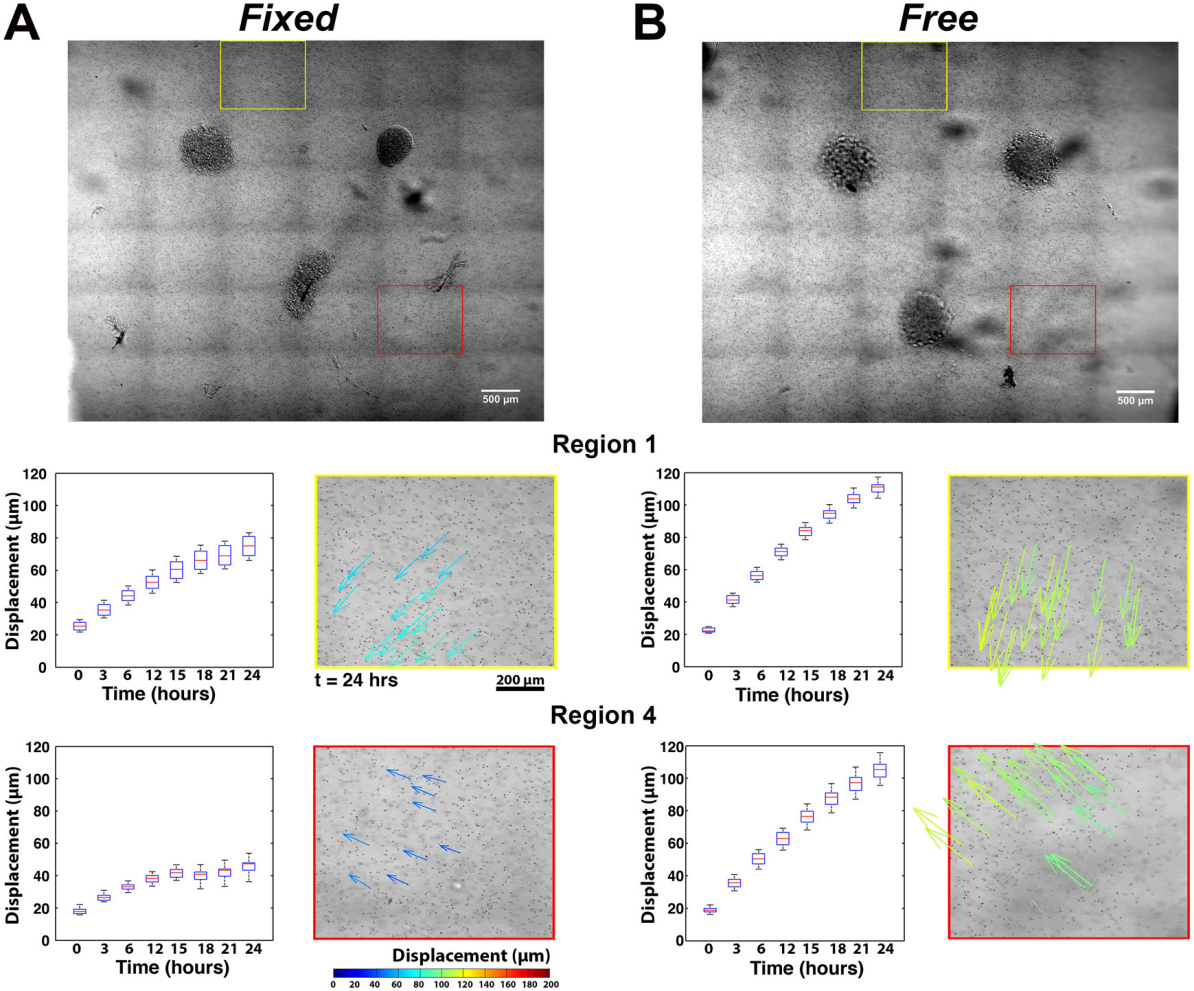
Comparison of Regional Differences in Microsphere Displacements. In both (A) *Fixed* and (B) *Free* gels the cumulative displacements of images associated with Region 1 (yellow box) and Region 4 (red box) at each hour are represented with box plots, where the box edges show the 25^th^ and 75^th^ percentiles, the median is depicted with a red line, and the whiskers indicate the full range of the data points. Color-coded arrows show the direction and magnitudes of the cumulative displacements at t = 24 hours. Microsphere displacements are directed towards either the explants or the axial regions between explants. Larger microsphere displacements occurred in the *Free* gel compared to the *Fixed* gel.

Significant regional differences in the microsphere displacements were also observed (Fig 5A). In both the *Free* and *Fixed* gel, microsphere displacements were statistically no different in the axial regions (Region 3) between explants, except at 24 hours. The average cumulative microsphere displacements in the non-axial regions (Regions 1, 2, and 4) were, however, significantly greater for the *Free* gel than for the *Fixed* gel at all time points. In the *Fixed* gel, displacements in the non-axial regions were essentially the same or slightly lower than in Region 3. A more detailed view of the microsphere displacements from Region 1 and Region 4 is presented in Fig 6. It shows that the cumulative microsphere displacements in both non-axial regions began to plateau noticeably after approximately 12 hours in the *Fixed* gels (Fig 6A), whereas for the *Free* gel, the displacements in these same regions increased roughly linearly before beginning to plateau later in the experiment (Fig 6B).

Microsphere displacement rates also differed regionally (Fig 5B). In the *Free* gel, period average rates of displacement were generally highest in the non-axial regions of the gel. At six hours, the rates were essentially equivalent, with values of 0.13 ± 0.05 μm/min, 0.13 ± 0.03 μm/min, and 0.13 ± 0.05 μm/min in Regions 1, 2, and 4, respectively. These rates decreased appreciably to 0.08 ± 0.03 μm/min, 0.07 ± 0.02 μm/min, and 0.09 ± 0.03 μm/min after 12 hours, and finally to 0.04 ± 0.01 μm/min, 0.03 ± 0.01 μm/min, and 0.05 ± 0.02 μm/min after 24 hours. Period average rates of displacement in the axial region (Region 3), were considerably smaller, with rates of 0.09 ± 0.02 μm/min after six hours, 0.06 ± 0.02 μm/min after 12 hours, and 0.03 ± 0.01 μm/min after 24 hours. In the *Fixed* gel, the period average rates of displacement were generally lower and also decayed faster with time in each region compared to the *Free* gel. The exception to this trend was in Region 3, where even though the rates were lower in the *Fixed* gel, no significant difference in the period average rates between *Fixed* and *Free* gels was found. Instantaneous rates of displacement generally followed the period average rates of displacement, but were initially higher with maximum measured rates of 0.17 ± 0.06 μm/min and 0.21 ± 0.06 μm/min occurring at *t* = 15 minutes for *Free* and *Fixed* gels, respectively.

**Fig 7:**
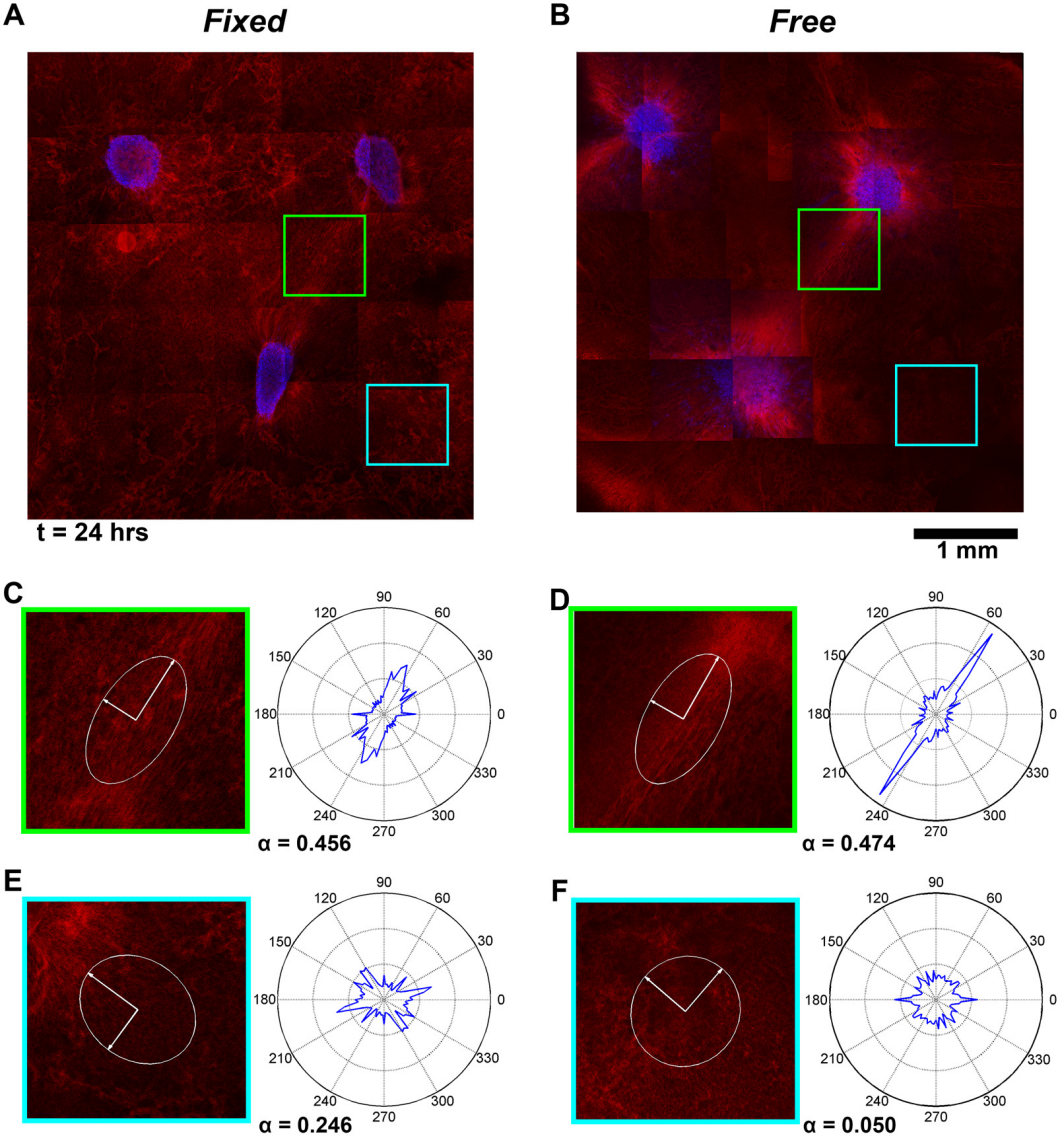
Fibrin fiber alignment in a *Fixed* and Free gel at t = 24 hours. Equivalent, microsphere free (A) *Fixed* and (B) *Free* gels were imaged with reflectance mode confocal microscopy and tiled together. Fiber orientation distributions for each image were reduced to major and minor principal directions of alignment and plotted at the center of each image as a pair of axes circumscribed by an ellipse. Highly aligned regions are more elliptical and less aligned regions are more circular. Fiber distributions for highly aligned axial regions (C, D) and less aligned, non-axial regions (E, F) are also shown.

Fibrin fiber realignment between explants was also apparent after 24 hours in reflection mode confocal images of microsphere-free *Fixed* and *Free* gels (Fig 7). The average strength of fiber alignment in the *Free* (α = 0.246 ± 0.132) and *Fixed* (α = 0.209 ± 0.096) gels were not significantly different. For both gels, fiber realignment was strongest in the axial regions between explants. Interestingly, strong fiber alignment did not develop equally among all three axes. Instead, one or two dominant axes of fiber alignment developed between the explants. In the *Fixed* gel, strong fiber alignment occurred between the bottom explant and the top-right explant (α = 0.456) (Fig 7C), and between the top two explants (α = 0.437). In the axis that developed the strongest alignment the final centroid-to-centroid distance was 1.97 mm, which was smaller than along the other two axes (2.32 mm and 2.38 mm). In the *Free* gel, strong fiber alignment was also observed between the top-right explant and the bottom explant (α = 0.474) (Fig 7D). Similar to the *Fixed* gel, the centroid-to-centroid distance along this axis (2.01 mm) was shorter than the other two axes (2.11 mm and 2.70 mm). Repeat experiments (data not shown) on additional samples confirmed this asymmetrical outcome in fiber alignment, although equal alignment between all three axes was also found in some gels. This result may be due to inherit asymmetries in the positioning of the explants, the number of cells in each explant, or other factors that drive preferential remodeling and fiber alignment along some axes. For both boundary conditions, increased radial fiber alignment was also observed around the explants, with the lowest levels of alignment found in non-axial regions of the gel (Fig 7E and 7F).

Finally, gel restructuring through the thickness was also observed indirectly from the amount of periodic refocusing required to keep tracked microspheres focused in the time-lapse images. Although it was not quantified directly, the *Free* gel required more refocusing than the *Fixed* gel (the focal plane was lowered 44 μm versus 18 μm, respectively, over 24 hours).

### Computational Model

**Fig 8:**
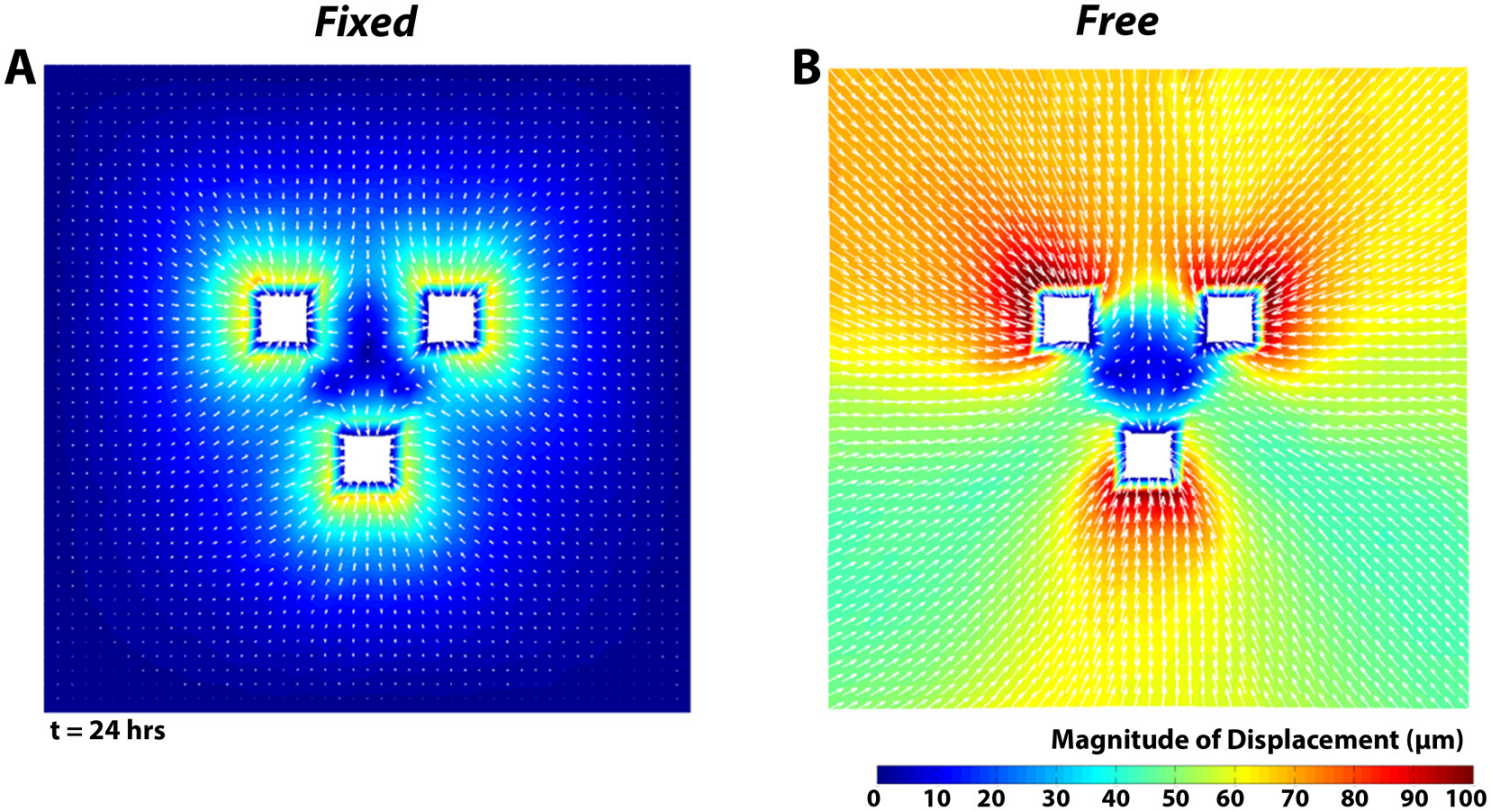
Model predictions of displacement. Displacement fields for (A) the *Fixed* and (B) *Free* conditions at 24 hours. White arrows indicate the direction of ECM displacement towards the compacting explants.

Model predictions for both *Fixed* and *Free* boundary conditions resulted in qualitatively similar morphological changes as in the experiment (Fig 8, S3 and S4 Movies). For the *Free* condition, the simulation was terminated when the final explant area shrank to 66% of its initial area to 0.319 ± 0.001 mm^2^, in close agreement with the experiment. For the same amount of simulated compaction, the explant area in the *Fixed* condition reduced to 69% of the initial area to 0.332 ± 0.002 mm^2^, also in close agreement with the experiment. The centroid-to-centroid distances shortened to 97.1% ± 0.2% and 99.2% ± 0.0% the initial distances in the *Free* and *Fixed* simulations, respectively, but these changes in distance were not as much as in the experiment.

Model predictions of displacements in the gel were also qualitatively similar to the experiments in several ways (Fig 8). First, the overall patterns of the displacement fields were similar, with the largest displacements occurring in regions closest to the explants. In addition, much larger displacements were predicted in the *Free* condition compared to the *Fixed* condition. But, the predicted regional average cumulative displacements (Fig 5C) were substantially lower than in the experiments (roughly 1.5 to 3.3 times lower at 24 hours). Even so, the relative regional differences in the average cumulative displacements predicted by the model for each boundary condition were strikingly similar to the experiment, with higher displacements in the non-axial regions than in the axial region in the *Free* gel, and higher displacements in the axial region than in non-axial regions in the *Fixed* gel. An additional notable difference between the model and experiment concerned the period average rate of microsphere displacement (Fig 5D). In the models, the rate continued to increase during the simulation in opposition to the decreasing rate observed in the experiment.

**Fig 9:**
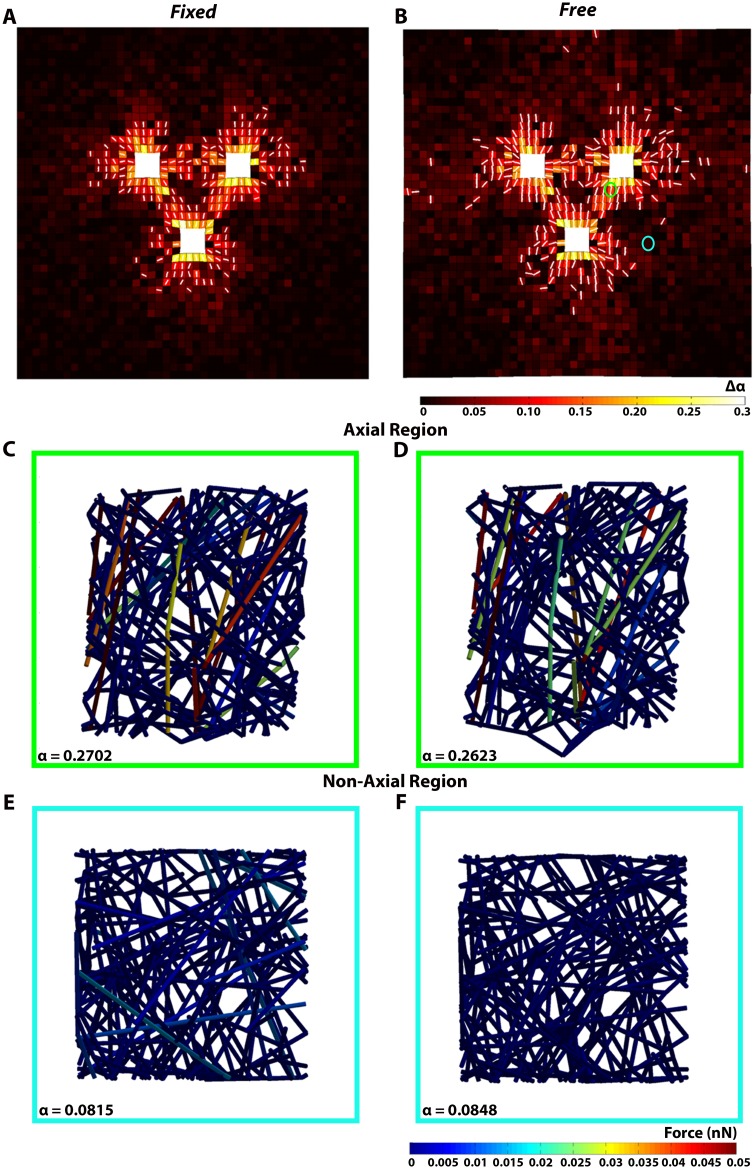
Model prediction of fiber realignment. Fiber network realignment at t = 24 hours for a (A) *Fixed* and (B) *Free* gel. The color map indicates the change in the strength of fiber alignment (Δ*α*) from the initial network configuration. Also shown as white arrows are the principal directions of fiber alignment for those elements where Δ*α* > 0.05. Network reorganization and fiber forces for a representative network in an axial (C,D) and non-axial region (E,F) in roughly equivalent locations as in Fig 7.

Model predictions of fiber network reorganization were also similar to those observed experimentally (Fig 9). For both *Fixed* and *Free* gels, changes in the strength of fiber alignment (Δ*α*) were greatest around the explants and in the axial regions between explants (Fig 9C and 9D). Fiber networks located away from the explants were mostly unaffected and remained close to isotropic in both conditions (Fig 9E and 9F). Although the fiber alignment patterns between the two simulations were similar, small differences were predicted. In the *Free* gel, more surface ECM elements had a change in the initial strength of fiber alignment Δ*α* > 0.05 than in the *Fixed* gel (281 versus 234, respectively). In addition, the overall average Δ*α* was slightly higher in the *Free* gel (0.0278 ± 0.0353) than in the *Fixed* gel (0.0216 ± 0.0357), but the overall *α* was the same at 0.1506 ± 0.0738 and 0.1505 ± 0.0736 in the *Free* and *Fixed* gel, respectively. In contrast, network fiber forces were higher in the *Fixed* gel (1.3 ± 8.6 pN) than in the *Free* gel (0.5 ± 6.3 pN). Also, even though the symmetry of the model resulted in strong axial alignment between all three explants, greater spacing between the top two explants than with the bottom explant resulted in significantly lower average fiber alignment strength (*α* = 0.134±0.061 and *α* = 0.133±0.063 in the *Fixed* and *Free* gels, respectively) than in the other axial regions (*α* = 0.279±0.080 and *α* = 0.268±0.055 in the *Fixed* gel, and *α* = 0.276±0.077 and *α* = 0.266±0.054 in the *Free* gel).

## Discussion

Tissue growth, remodeling, and repair are complex processes that are regulated in part by multi-scale mechanical interactions. To better understand how these interactions contribute to the mechanobiology of the remodeling process in the context of wound healing and scar formation, we conducted *in vitro* experiments on fibroblast-matrix interactions in fibrin gels (as an approximation of the initially formed clot) subjected to either *Free* or *Fixed* in-plane mechanical constraints. Using averaged morphological measurements from the experiments, an idealized, image-based multi-scale model was used to simulate the experiment so that the ability of the model to capture the physics of short-term structural remodeling could be evaluated.

Experimentally, we found that the explant system produced significant differences in explant morphology and fibrin gel reorganization in a manner that depended on whether the lateral edges of the gel were mechanically constrained or not. For the *Fixed* gel the average reduction in explant area and centroid-to-centroid distance was less than in the *Free* gel. Furthermore, much larger microsphere displacements and greater compaction through the thickness were observed in the *Free* gel compared to the *Fixed* gel. In addition, regional cumulative displacement patterns and rates of displacement differed in the non-axial regions (but not the axial regions) of the two gels. Taken together, these data imply that there was less “slack” (i.e., translational and rotational freedom) in the fibrin fiber networks for the *Fixed* gel, a result consistent with other studies involving the role of mechanical constraints on fibrous gels [27,50–52]). In these studies, cooperative behavior between cell tractions and fiber reorganization resulted in anisotropic gel reorganization that proceeded in a manner that was dependent on the gel geometry, gel boundary conditions, and the spatial patterning of the cells. We interpret the differences in constrained and unconstrained gels in this study as a product of multi-scale mechanical interactions that arise from such cooperative behavior. Initially, the fibroblasts pulled fibrin fibers inward to produce a dense, pericellular matrix around the explants. Distal fibers then rotated and translated inward in response to tensile forces that were propagate through the fiber network. The extent of translation and rotation depended on the existence and distance of mechanical constraints, which consisted of either the in-plane gel attachments to the boundaries of the PDMS mold and glass substrate, or the cell tractions emanating from the explants. Instead of translating inward, constrained fibers, rotated, stretched, and aligned along directions of tension based on their connectivity to other simultaneously deforming fibers in the gel. This collective, multi-scale response produced fiber alignment patterns that then set the pattern for cell migration.

In both *Fixed* and *Free* gels, cell migration distance (*CMD*) in the axial regions between explants was significantly greater than migration into non-axial regions. This finding is consistent with the concept of contact guidance, where cells preferentially migrate along directions of fiber alignment, and has been observed by others in other cell-gel systems [38,51,53]. For example, Provenzano et al. saw that radial fiber alignment generated by explants enhanced and directed cell migration out of the explants, and then postulated that this mechanism might underlie metastasis into the surrounding stroma for certain cancers [38]. However, it is also possible that the fibroblasts are migrating faster between explants due to a durotactic effect because the aligned fibers are stiffer [54], or to direct sensation of tension from the explant [51], or to a ligand density-dependent haptotactic effect [55], or to some combination thereof. Chemical gradients could also be involved, but the fact that *CMD* in non-axial regions was significantly greater in the *Fixed* condition than in the *Free* condition suggests that a mechanical effect was more likely.

**Fig 10:**
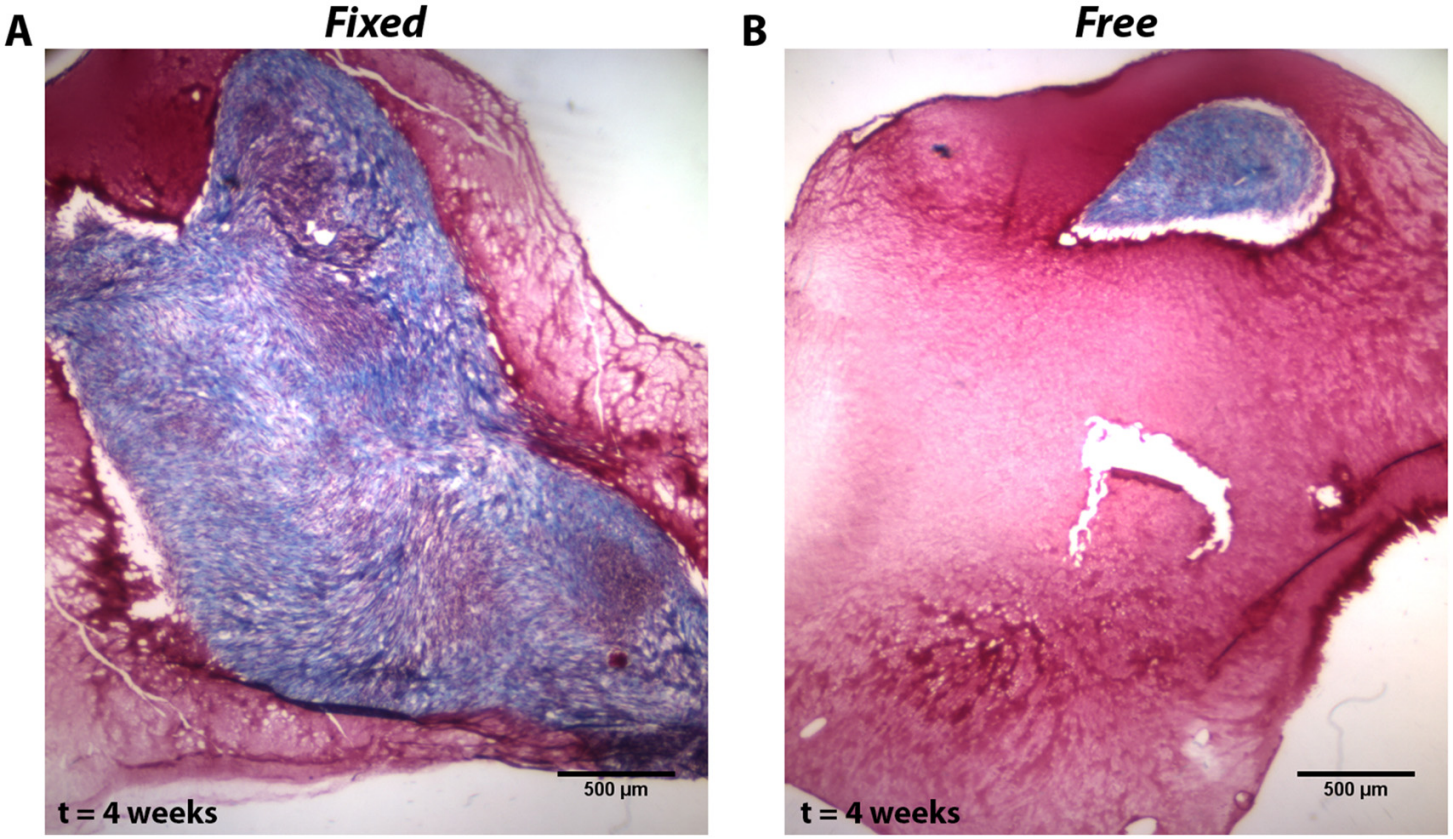
Pilot Study of Compositional Remodeling after 4 weeks of culture. Mason’s trichrome stain for a (A) *Fixed* and (B) *Free* gel after 4 weeks of culture supplemented with TGF-β1 and ascorbic acid. More collagen (blue) was produced in *Fixed* gels than in *Free* gels.

Regardless of the exact mechanism(s) involved, the anisotropy in fiber alignment and cell migration that develops in the fibrin gel should control how ECM remodeling progresses, particularly with respect to the organization and amount of collagen produced [26]. To provide a preliminary indication of this possibility, we performed a simple pilot study to assess whether substantive differences in long-term remodeling occur between the *Fixed* and *Free* gels even though only small differences in short-term remodeling exist. Gels were cultured for four weeks and subjected to identical conditions with one exception: 50 μg/mL of ascorbic acid and 1 ng/mL of TGF-β1 was also added to the medium to enhance collagen production [26]. Mason’s trichrome staining qualitatively demonstrated considerably more collagen production in *Fixed* gels than in *Free* gels (Fig 10). In related work, John et al. [56] found that stiff boundaries resulted in more α-smooth muscle actin (α-SMA) expression in fibroblast populated collagen gels compared to compliant boundaries, and that this expression was enhanced with the addition of TGF-β1. α-SMA is a hallmark of the myofibroblast phenotype, which is associated with increased collagen production and abnormal scarring [57]. Although we did not look for α-SMA in this pilot study, it seems likely that the increase in collagen observed in the *Fixed* gels will be also associated with increased α-SMA. More work will be required to quantify these and other differences in ECM synthesis and degradation and how they link to fibroblast mechanotransductive pathways, such as Rho/Rock [38] and ERK [25,58], particularly as we explore how manipulating the mechanical environment at various time points could be used to control the remodeling process.

The *in vitro* system presented here is, out of necessity, an approximation of the complex and dynamic composition and organization of the ECM in a human cutaneous wound. As a starting point for developing this *in vitro* system, we elected to use 6.8 mg/mL fibrin gels made from bovine fibrinogen because we have prior experience working with these gels [26], and they are well characterized for their use in several tissue engineering applications [25,29,34,59]. Fibrin is the most important component of the provisional ECM at the wound site. It should be noted, however, that these gels are much simpler in composition and microstructure compared to the provisional fibrin matrix of a human clot, which also consists of an assortment of plasma proteins, cytokines, growth factors, enzymes, and activated platelets [60]. Reported ranges for the concentration of fibrinogen in human blood typically span from 1.5 to 4.5 mg/mL [61]. Since the provisional fibrin matrix of a clot increases in density, first as activated platelets exert tractions forces on it, and then later, when fibroblasts migrate into and remodel it, the 6.8 mg/mL density of these gels can be seen as a physiologically relevant, though temporal, gel concentration for this system. However, it is also important to realize that a number of interdependent factors can dramatically affect fibrin microstructure and organization during *in vitro* gel reconstitution, such as pH, ionic strength, temperature, fibrinogen and thrombin ratios and concentrations, and the presence of other plasma proteins [60,62–65]. Other factors present *in situ* during clotting will also likely present a provisional matrix that differs from the fibrin gel used in this model. Consequently, all of these variables should be taken into account when drawing conclusions about how the results from this *in vitro* system compare to human cutaneous wound healing. Future modifications to the system to make it more like the wound site could include the addition of plasma proteins and other cell phenotypes, such as platelets, keratinocytes, and leukocytes.

With regard to the modeling component of this study, the image-based computational models were able to predict boundary condition dependent relative changes in explant morphology and spacing, regional differences in microsphere displacements, and fiber alignment patterns that qualitatively matched what was observed experimentally. However, the model predicted much lower displacement magnitudes and an increase in the period average rates of displacement over time instead of a decrease. Small differences in fiber alignment between the *Free* and *Fixed* gels were also predicted, but the imaging method used did not clearly show whether such differences in alignment between gels existed or not. Instead, these images showed stronger alignment in axial regions between explants than in non-axial regions, and that one or two dominant axes of fiber alignment formed instead of three, regardless of the boundary conditions. As stated earlier, formation of a dominant axis of alignment in the experiments could be a result of asymmetries in the experiment, such as the centroid-to-centroid distance or the size/number of cells, and thus the total force associated with each explant. The model did predict less alignment in the matrix between the top two explants, which were farther apart from each other than with the bottom explant, than between each of the top explants and the bottom one, which is consistent with our experimental observations that dominant axes form between explants that are closer together.

Several model refinements could potentially improve model predictions. For one, the description of the microstructure could be improved to better reflect the micromechanics of fibrin fibers in the gel. Currently, network fibers are represented as single segments that are cross-linked to other fibers via ball joints that cannot resist moments. Others have represented network fibers as a series of connected segments with torsion springs at the joints that resist rotation [66–68]. This representation allows for curved fibers that can collapse locally yet still transmit tension as they are gathered into a pericellularly dense region around the explants. Some of these models have reproduced this densification and the development of alignment between pairs of cells [67,68]. Incorporating such behavior into our model should increase the predicted cumulative displacements and also help match the fibrin densification observed experimentally.

Another area requiring improvement is in modeling the manner in which cells generate and respond to forces. In the model, cell traction forces are generated by incrementally shortening the reference length of cellular network fibers the same amount at each step. This approach does capture the alignment produced and relative differences in cumulative displacements, but predicted rates of displacement continued to increase over time, which is in opposition to the experimentally observed decrease with time. This behavior suggests that the cells are modulating how much they deform the surrounding matrix based on either a feedback-controlled, force-sensing mechanism, or possibly by saturation of the number of cell-ECM binding sites [69]. Others have modeled the development of traction forces with the former mechanism in mind [70,71]. For example, Wang et al. incorporated the well-known Hill-relation used to describe muscle contraction [72]. We anticipate that incorporating similar changes into the model will result in a decrease in the rate of displacement with time and better predict the short-term remodeling observed experimentally. Finally, although it was beyond the scope of this work, a mechanism for degrading the fibrin fibers and replacing it with collagen fibers will also be necessary in order to predict long–term remodeling and propensity for scarring.

## Conclusions

Multi-scale mechanical interactions in wound healing are poorly understood. They are particularly important to characterize because they control both the manner in which mechanical signals are propagated to the cellular level to direct cell activity and the ultimate mechanical behavior and function of the healed tissue. Due to the complexity of these processes, it is necessary to develop computational models that serve as a theoretical basis for predicting how ECM remodeling proceeds. In this study, we began the development of this kind of modeling system by quantitatively characterizing small but significant differences in an *in vitro* explant-fibrin gel system with *Fixed* and *Free* in-plane mechanical constraints. We also performed a rigorous comparison with an image-based multi-scale mechanical model and found qualitative agreement with the experiments in some areas. Discrepancies between model and experiment provide fertile ground for challenging model assumptions and devising new experiments to enhance our understanding of how this multi-scale system functions. These efforts will ultimately improve the predictions of the remodeling process, particularly as it relates to dermal wound healing and the reduction of patient scarring. Such models could then be used to recommend patient-specific mechanical-based treatment dependent on parameters such as wound geometry and location and patient age and health.

## Supporting Information

S1 Fig Tiles analyzed for bead displacement. Individual tiles stitched to generate a larger image of the fibrin gel surface were analyzed individually to quantify microsphere displacements in each condition. Tiles are color coded to indicate the region of the gel to which they correspond. Tiles surrounded by a yellow box correspond to Region 1, tiles in blue correspond to Region 2, tiles in green correspond to Region 3, and tiles in red correspond to Region 4.
(DOCX)

S1 Movie Time-Lapse of explant remodeling in the *Free* fibrin gel. Time-lapse images of the tiled 6x6 imaging area for the *Free* fibrin gel over 24 hours. Color-coded arrows represent the magnitude and direction of cumulative microsphere displacements.
(AVI)

S2 Movie Time-lapse of explant remodeling in the *Fixed* fibrin gel. Time-lapse images of the tiled 6x6 imaging area for the *Fixed* fibrin gel over 24 hours. Color-coded arrows represent the magnitude and direction of cumulative microsphere displacements.
(AVI)

S3 Movie Model predictions of displacement in a *Free* fibrin gel. Model predictions for the displacement fields in the *Free* condition are shown in microns. White arrows also indicate the direction of displacement.
(AVI)

S4 Movie Model predictions of displacement in a *Fixed* fibrin gel. Model predictions for the displacement fields in the *Free* condition are shown in microns. White arrows also indicate the direction of displacement.
(AVI)
