# Supplementary material for: A Combined In Vitro Imaging and Multi-Scale Modeling System for Studying the Role of Cell Matrix Interactions in Cutaneous Wound Healing
Source: PLoS One. 2016 Feb 3;11(2):e0148254. doi: 10.1371/journal.pone.0148254 (PMC4739727; doi:10.1371/journal.pone.0148254)
Supplement: S1 Fig — Individual tiles stitched to generate a larger image of the fibrin gel surface were analyzed individually to quantify microsphere displacements in each condition. Tiles are color coded to indicate the region of the gel to which they correspond. Tiles surrounded by a yellow box correspond to Region 1, tiles in blue correspond to Region 2, tiles in green correspond to Region 3, and tiles in red correspond to Region 4. (DOCX) [file pone.0148254.s001.docx]

**Supporting Information**

**Table S1. Microsphere Average Displacements at 24 hours**

|  | **Fixed** | | **Free** | |
| --- | --- | --- | --- | --- |
| **Image Location** | **Experimental (µm)** | **Computational (µm)** | **Experimental (µm)** | **Computational (µm)** |
| **1** | 54.2 +/- 8.0 | 12.3 +/- 4.3 | 51.7 +/- 5.3 | 72.9 +/- 2.0 |
| **2** | 46.1 +/- 4.3 | 17.9 +/- 7.0 | 85.6 +/- 8.4 | 72.7 +/- 3.6 |
| **3** | 68.0 +/- 14.8 | 16.8 +/- 6.6 | 101.2 +/-4.6 | 64.6 +/- 4.4 |
| **4** | 63.7 +/- 8.1 | 12.5 +/- 4.2 | 73.8 +/- 7.2 | 54.0 +/- 3.6 |
| **5** | 52.0 +/- 7.8 | 8.2+/- 2.6 | 63.2 +/- 3.8 | 46.5 +/- 1.2 |
| **6** | 39.2 +/- 5.2 | 5.4 +/- 1.7 | 52.1 +/- 4.6 | 46.4 +/- 1.1 |
| **7** | 77.2 +/- 7.2 | 24.9 +/- 7.8 | 84.1 +/- 8.0 | 77.5 +/- 5.2 |
| **8** | 109.8 +/- 18.1 | 50.8 +/- 10.4 | 110.8 +/- 6.1 | 95.3 +/- 8.5 |
| **9** | 106.3 +/- 9.3 | 42.8 +/- 11.0 | 104.2 +/- 9.3 | 68.1 +/- 13.7 |
| **10** | 82.9 +/- 6.3 | 26.0 +/- 4.8 | 94.4 +/- 4.5 | 51.1 +/- 4.4 |
| **11** | 72.6 +/- 6.0 | 19.2 +/- 5.4 | 89.8 +/- 5.1 | 49.8 +/- 4.4 |
| **12** | 63.3 +/- 7.8 | 12.7 +/- 4.1 | 98.8 +/- 17.5 | 54.8 +/- 4.3 |
| **13** | 74.8 +/- 6.3 | 23.7 +/- 6.6 | 111.2 +/- 3.9 | 71.0 +/- 3.5 |
| **14** | 100.6 +/- 9.9 | 39.7 +/- 14.5 | 89.6 +/- 11.6 | 64.4 +/- 20.2 |
| **15** | 86.4 +/- 6.3 | 23.8 +/- 14.0 | 78.2 +/- 4.9 | 21.0 +/- 11.7 |
| **16** | 87.0 +/- 24.3 | 30.9 +/- 12.4 | 64.6 +/- 15.9 | 41.1 +/- 15.4 |
| **17** | 121.9 +/- 6.8 | 47.3 +/- 11.7 | 128.4 +/- 15.7 | 77.2+/- 13.2 |
| **18** | 97.4 +/- 3.3 | 25.2 +/- 7.7 | 198.3 +/- 9.2 | 69.1 +/- 4.6 |
| **19** | 72.0 +/- 5.5 | 24.5 +/- 7.0 | 123.6 +/- 9.0 | 69.7 +/- 3.4 |
| **20** | 56.1 +/- 21.9 | 35.8 +/- 15.5 | 100.4 +/- 41.6 | 61.7 +/- 19.3 |
| **21** | 47.5 +/- 16.9 | 19.4 +/- 12.5 | 77.9 +/- 10.6 | 20.1 +/- 9.9 |
| **22** | 91.7 +/- 14.7 | 31.5 +/- 12.1 | 97.5 +/- 8.6 | 38.1 +/- 15.2 |
| **23** | 97.9 +/- 11.7 | 48.9 +/- 11.6 | 136.3 +/- 16.5 | 76.8 +/- 14.3 |
| **24** | 94.5 +/- 9.0 | 25.9 +/- 8.1 | 170.3 +/- 15.3 | 69.1 +/- 5.2 |
| **25** | 74.7 +/- 8.4 | 24.8 +/- 8.0 | 135.2 +/- 8.4 | 72.3 +/- 5.2 |
| **26** | 128.8 +/- 8.0 | 49.8 +/- 10.6 | 171.9 +/- 11.2 | 90.0 +/- 8.5 |
| **27** | 76.2 +/- 12.4 | 44.1 +/- 10.5 | 150.2 +/- 9.7 | 67.6 +/- 12.7 |
| **28** | 59.9 +/- 4.0 | 26.1 +/- 5.8 | 120.8 +/- 6.9 | 49.7 +/- 4.4 |
| **29** | 47.4 +/- 3.6 | 18.7 +/- 5.4 | 105.9 +/- 5.6 | 48.2 +/- 4.3 |
| **30** | 56.9 +/- 7.4 | 13.1 +/- 4.3 | 105.5 +/- 5.1 | 53.4 +/- 4.2 |
| **31** | 53.2 +/- 8.8 | 12.1 +/- 4.2 | - | 67.0 +/- 2.6 |
| **32** | 82.4 +/- 10.9 | 17.5 +/- 6.9 | - | 66.6 +/- 3.8 |
| **33** | 61.8 +/- 7.3 | 17.2 +/- 6.6 | - | 60.5 +/- 3.9 |
| **34** | 44.3 +/- 3.7 | 12.8 +/- 4.1 | - | 53.0 +/- 3.0 |
| **35** | 30.1 +/- 3.2 | 7.8 +/- 2.2 | - | 45.0 +/- 1.2 |
| **36** | 23.5 +/- 2.1 | 5.3 +/- 1.6 | - | 45.2 +/- 1.3 |

**
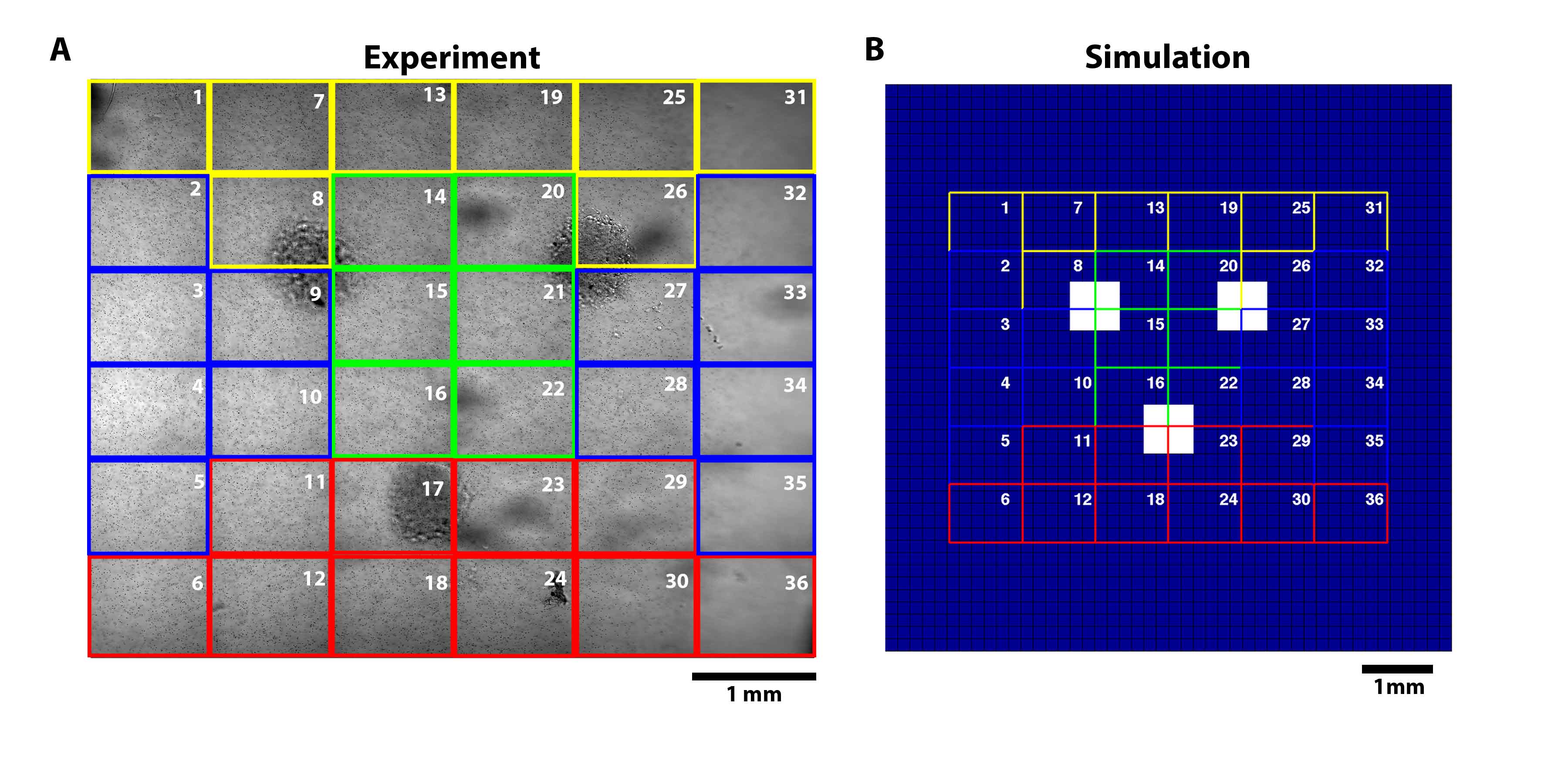
**

**Figure S1**.

.
